# Influence of Microsite Disturbance on the Establishment of Two Congeneric Invasive Thistles

**DOI:** 10.1371/journal.pone.0045490

**Published:** 2012-09-18

**Authors:** Emily S. J. Rauschert, Katriona Shea

**Affiliations:** 1 Department of Biology, St. Mary’s College of Maryland, St. Mary’s City, Maryland, United States of America; 2 Intercollege Graduate Degree Program in Ecology, Department of Biology and Department of Crop and Soil Sciences, The Pennsylvania State University, University Park, Pennsylvania, United States of America; 3 Department of Biology and Intercollege Graduate Degree Program in Ecology, The Pennsylvania State University, University Park, Pennsylvania, United States of America; Brigham Young University, United States of America

## Abstract

The successful establishment of invasive species has been shown to depend on aspects of the invaded community, such as gap characteristics. Biotic resistance may be particularly critical for stopping invaders at early life history stages, but new species can often invade following disturbances, which may create microsites with very different characteristics than are usually present. We examine the response of two invasive thistle species, *Carduus nutans* L. and *C. acanthoides* L., to three different microsite characteristics: disturbance type, size, and water availability. The two species initially responded differently to the type of disturbance: *C. acanthoides* had higher emergence and survival in plots with both above- and belowground disturbance, whereas *C. nutans* had better early performance in large microsites with above-ground disturbance only. Later in their life cycle, *C. nutans* performed better in plots that had been disturbed both above- and belowground, whereas *C. acanthoides* was largely unaffected by disturbance type. Increased emergence and survival, larger size and a higher proportion flowering were observed in larger gaps for both species throughout the life cycle. Watering had a negative impact on *C. nutans* emergence and fall survival and on *C. acanthoides* survival to the following summer. Overall, these results suggest that disturbance-generated microsite characteristics (disturbance type and size) may have large impacts on establishment of these two *Carduus* species, which in turn may persist well beyond the initial stages of growth. Studying invader responses to disturbance can help us to understand under what circumstances they are likely to establish and create persistent problems; avoiding or ameliorating such situations will have significant management benefits.

## Introduction

A suitable microsite for emergence and seedling establishment is a necessary condition for the success of a plant. Gaps in the existing vegetation, occurring due to death of individuals, through animal activity or other disturbances, create opportunities for plants to establish due to the exposure of soil, removal of competing vegetation and removal of litter [1]. Characteristics of gaps can be important to the regeneration niche, which includes dispersal, germination, establishment and seedling development [2]. Gap properties, such as size, can have a large impact on plant establishment, growth and reproduction [3]. Although plants in larger gaps have fewer potential competitors, they are also more vulnerable to desiccation, leading to the potential for trade-offs in optimal gap sizes.

Better plant growth in gaps may also be because of a reduction in the intensity of belowground competition, and not just due to light availability, as root biomass has been shown to be lower in gaps in an abandoned hayfield [4]. Furthermore, the effects of above-ground and belowground competition are not necessarily additive; Cahill [5] found that for two rosette forming species, the combined competitive response to above-ground and below-ground competition were subadditive (i.e. the total response was less than the sum of the separate responses), indicating that these responses are not independent. As disturbances generally occur above ground only, or both above and belowground, an understanding of both is required to predict species responses. Even among species with generally similar life histories, species may have very different responses to gap opportunities [6].

Understanding the effect of microsite characteristics has particular importance in the case of invasive species, as biotic resistance is an important early barrier to invasion [7]. When resident species do not keep resource levels uniformly low, opportunities for species to invade may exist [8]. Disturbances can alter such opportunities, and are well-known to affect species diversity [9], [10], [11]. Fluctuations in the abundance of competitors, which affects the availability of space and light, can make the invasibility of a community quite dynamic, and communities previously resistant to invasion may suddenly be vulnerable following disturbance such as drought [12]. Natives may actually be more competitive under conditions of reduced resource (light, water or nutrient) availability, but invasives may be favored in areas of high resource availability [13].

In this study, we address the effect of microsite characteristics (in terms of whether only above-ground, or both above- and below-ground disturbance has occurred, gap size and water availability) on emergence and establishment of two non-native invasive plant species. *Carduus nutans* and *C. acanthoides* are invasive species of Eurasian origin which have spread and become invasive throughout the world [14]. For both *Carduus* species, we examined two cohorts (sown in consecutive years) and evaluated invader performance in terms of emergence, growth, and flower production to assess the effects of microsite characteristics (size, above/belowground disturbance and water availability) on distinct life history stages. We hypothesize that thistle performance will be best in plots that receive both above-ground and below-ground disturbance (decreasing both light and root competition), that emergence will be highest for both species in larger microsites (with fewer competitors) and that watering will have a beneficial impact (as this is a critical resource).

## Methods

### Study Species

*Carduus nutans* and *C. acanthoides* are herbaceous monocarpic perennials; they are sometimes annual, winter annual or perennial [15]. *Carduus nutans* seeds are larger (2–4 mm) and heavier (4 mg) than those of *C. acanthoides* (1–3 mm, 2 mg) [16]. When sown into intact (undisturbed) vegetation, both species have extremely poor germination [17]. Seeds require good contact with the soil in order to absorb enough moisture for germination, and both species have maximal emergence at depths of 0.5–1.0 cm in the soil [16]. *Carduus acanthoides* appears to be more sensitive to moisture stress, although *C. nutans* is also affected by moisture conditions [16]. Despite substantial propagule pressure, *C. nutans* seldom establishes in well-managed pastures in its invaded range, presumably due to a lack of suitable gaps for colonization [18]. For these species, emergence and early establishment has been shown to be a critical determinant of population growth [19]. Thus we also follow individuals from germination through to flowering, as the conditions favoring establishment may not benefit later life history stages [20].

*Carduus nutans* and *C. acanthoides* emergence and survival were previously shown to be microsite size-dependent in a one year study, although, surprisingly, a watering treatment appeared to have no effect on thistle emergence and survival [21]. It is also known that disturbance can enhance their emergence [17], [22], [23]. However, it has been suggested for *C. nutans*, despite the positive effects of disturbance generally, that there may be decreased emergence in large (3 m^2^) plots [24].

### Experimental Design

The experiment was conducted in a field at the Russell E. Larson Agricultural Center at Rock Springs, (40.711, −77.942) in Pennsylvania’s Ridge and Valley physiographic province, USA. The site is a former pasture that has been left ungrazed for more than a decade, with mostly weedy grasses and dicots present. The site is typical of a central Pennsylvanian hayfield; the dominant species are mostly non-native and include the grasses *Elymus repens, Arrhenatherum elatius, Dactylis glomerata* and *Phleum pratense*, and the dicots *Plantago lanceolata, Taraxacum officinale, Trifolium repens, Trifolium pratense* and *Galium* species. The seeds were collected from naturalized *Carduus* populations from areas that only have one species present (near Carlisle, PA for *C. nutans*, and in State College for *C. acanthoides*). Flower heads were dissected to remove seeds, after which seeds were sifted with mesh screens to remove small flat seeds with no embryo, which are typically not viable (E. Leichtman, unpublished data) [25].

Two independent cohorts were initiated in separate plots, one in the fall of 2004 and one in the fall of 2005. The experiment had a randomized, full block design with three treatments: watering, microsite size and type of microsite disturbance. There were a total of 32 plot types (2 species × 2 watering types × 4 microsite sizes × 2 microsite disturbance types), replicated ten times in each of the two cohorts (640 plots total).

Disturbances were created in two different ways: a clipping treatment disturbed only the above-ground biomass but left the soil intact, and a full disturbance treatment disturbed both above and below-ground areas. All plots were first clipped, and then the full disturbance plots received an additional belowground disturbance. Clipping involved removing all above-ground biomass with electric clippers or scissors; this is similar to the effect of close mowing or grazing, both of which are experienced in the pastures and roadsides where these species are common. The full disturbance treatment consisted of an above- and below-ground disturbance created by either roto-tilling the plot (for 30 and 50 cm edge length disturbances) or drilling holes in the soil with a bulb planting attachment (for the 5 and 15 cm disturbances), to an approximate depth of 10 cm. This treatment mimics disturbance in recently tilled areas, in marginal areas such as construction sites, and due to animal digging.

Four microsite sizes were investigated: squares with lengths of 5, 15, 30 and 50 cm along an edge. These sizes were chosen to span the range from the minimum size believed necessary for seedling establishment (K. Shea, unpublished data) through to the largest area we had observed being occupied by single large adults. Half of the plots received a watering treatment, which consisted of adding 1 L of water over a 50×50 cm area twice weekly. The amount of precipitation in the northeastern United States is predicted to increase under climate change, but there is a large amount of uncertainty in the amount of increase [26], [27], [28]; the amount added was intended to loosely mimic that projection while still falling within the range plants might experience in a wet year.

Four seeds of a species were sown in the soil at approximately 5 mm below the surface in the center of each disturbance in marked locations in September of 2004 (cohort 1) or 2005 (cohort 2). This controls for seed limitation and allows us to focus on the effects of microsite characteristics. After planting, disturbed plots were lightly hand-weeded to maintain differences between the treatments during the emergence period (i.e. for the first month). The watering treatment was applied from sowing in September until the end of the fall growing season in November during the first growing season of each cohort. Plots were censused three times weekly during the first month; all new emergences and the survival of previously emerged seedlings were recorded. Monitoring of bare plots next to the experimental sites resulted in no germinations of either species, thus all germination observed was attributed to the seeds sown. In November, the longest leaf length and diameter of each rosette was recorded, in addition to whether or not the plant experienced herbivory.

After overwintering, in May and June of 2005 and 2006, the longest leaf length and diameter of rosettes were measured. All mature flowerheads were bagged with pollen bags after pollination had occurred, to avoid further thistle seed contamination of the soil. In July 2005, 2006 and 2007, a destructive census of flowering plants was carried out in existing cohorts, as individuals senesce and die after flowering. The number of flower heads was recorded for flowering plants, and the longest leaf length was measured for rosettes. If a plot still contained a rosette, it was monitored in subsequent years, as in both cohorts, some individuals did not flower in their first, or even their second, summer. By 2007, however, nearly all plants had flowered or died, and the experiment was terminated.

### Statistical Methods

Analyses were performed separately for the two species. In order to avoid pseudoreplication, the average plot response was used as the response variable in all analyses [29]. We examined logistic regressions of the proportion of seeds planted that emerged and survived in the fall, the proportion of emerging individuals that survived to the following summer, and the proportion of individuals that flowered. In each case we used a vector of the number of successes to failures in order to account for sample size in estimating proportions [29]. We also examined linear regressions of log transformed size data (longest leaf length at the end of fall and spring).

The potential explanatory variables were: whether or not a plot was watered, the relative area of the microsite disturbance (scaled to the largest disturbance area, to allow easier comparison with other variables) and the type of the microsite disturbance. All variables were centered and standardized following Schielzeth [30].

Generalized linear mixed effect models were fit using the lmer package [31] in R [32], using block and cohort as random effects. Likelihood ratio tests were used on nested models to determine whether including interactions between treatments significantly improved model fit [33]. If the best model contained higher order interactions, models were refitted on subgroups of the data. *P*-values were estimated using likelihood ratio tests to compare models with and without the parameter of interest; models were fit using maximum likelihood.

## Results

Microsite characteristics strongly influenced the establishment of both species. Overall, *C. nutans* had higher emergence rates than *C. acanthoides* (22% and 39% for *C. nutans* and 13% and 24% for *C. acanthoides* in the two cohorts). Nearly all emergence occurred in the fall; in 2005 only four new emergences were observed in the spring for the cohort planted in 2004. *Carduus nutans* grew to slightly larger sizes than *C. acanthoides* by early November of the first season in both cohorts. A higher proportion of *C. nutans* plants present flowered in their first year (7.34% for *C. nutans* versus 1.25% for *C. acanthoides*), with similar numbers flowering in the second year (approximately 24% for both species).

**Figure 1:**
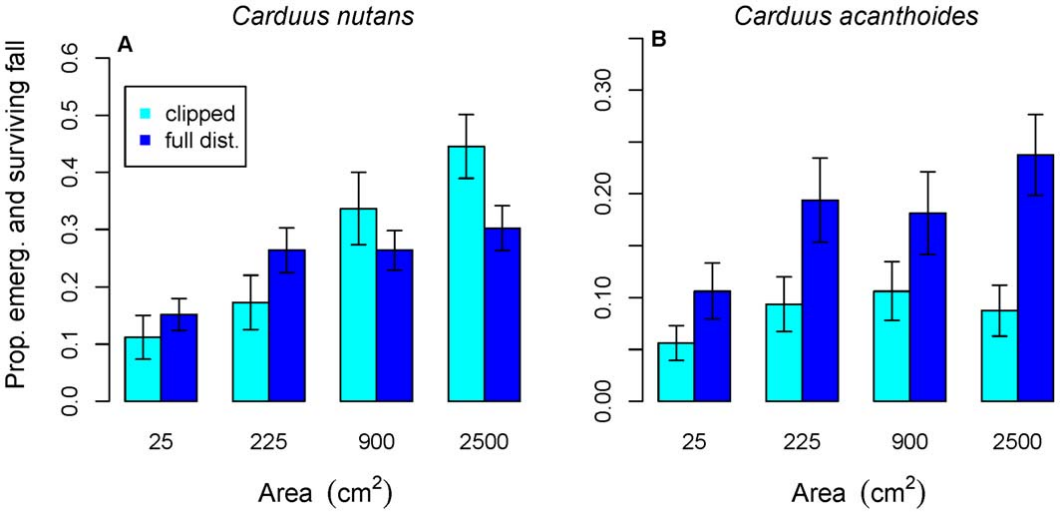
Effect of microsite area on fall emergence and survival of *Carduus nutans* and *C. acanthoides.* Panels a and b show the combined emergence and survival response for *C. nutans* and *C. acanthoides* (darker blue indicates full disturbance, lighter bars indicate clipped areas). Emergence and survival are generally higher in larger microsites. For *C. acanthoides,* performance was better in fully disturbed microsites. *Carduus nutans* did better in larger clipped microsites but smaller fully disturbed microsites.

**Figure 2:**
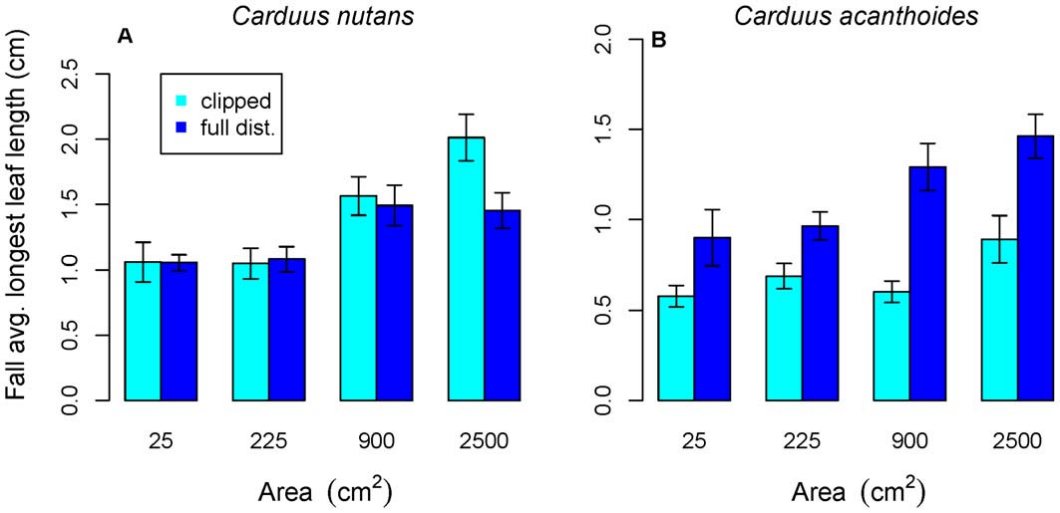
Effect of microsite area and type of disturbance on rosette size in fall. Panels a and b show that rosettes were larger in larger microsites for both species in the fall (darker blue indicates full disturbance, lighter bars indicate clipped areas). For *Carduus acanthoides,* rosettes are larger in fully disturbed plots.

**Figure 3:**
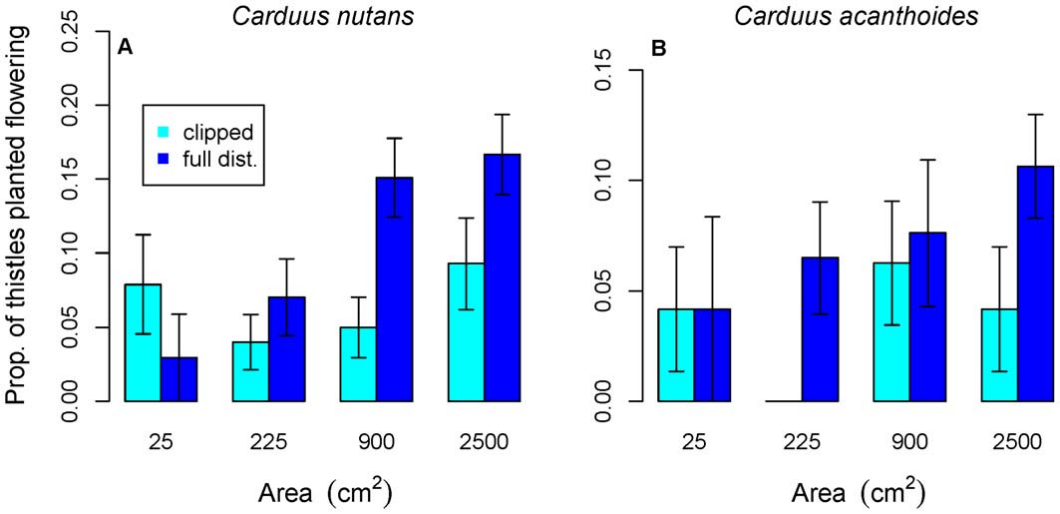
The response of the proportion of thistles present to microsite size. Both species were more likely to flower in larger microsites (darker blue indicates full disturbance, lighter bars indicate clipped areas). For *C. acanthoides*, the effect of disturbance type is not significant. No *C. acanthoides* individuals flowered in clipped 225 cm^2^ microsites.

*Carduus nutans* initially performed better (higher germination and survival rates and larger size) in large microsites in clipped versus fully disturbed plots (Fig. 1a, Fig. 2a), but later had better performance (larger size, more flowerheads produced and a higher proportion flowering) in fully disturbed plots (Fig. 3). For *C. nutans,* the sign of the interaction term in the model of emergence and fall survival of fully disturbed plots was positive, in contrast to clipped plots, where it was negative. Microsites with both watering and larger area had higher germination and survival rates in clipped plots, but this combination led to lower germination and survival rates in fully disturbed plots. *Carduus acanthoides* had better performance in fully disturbed plots (Figs. 1b, 2b), although this is not significant for survival to July or the proportion flowering (Fig. 3b).

Larger microsite size was significantly positively associated with all aspects of *C. nutans* performance except survival to July in clipped plots (Fig. 1–3, Tables 1 and 2). For *C. acanthoides,* the models also consistently show a significant positive effect of microsite size. Microsite area had a larger impact on *C. nutans* emergence and fall survival in fully disturbed plots than in clipped (above-ground disturbance only) plots.

Emergence and fall survival in clipped plots had a negative response to the watering treatment (Table 1) for *C. acanthoides* and for *C. nutans* in clipped plots. There was no significant size response (average longest leaf length) to watering for either species. By the spring (Table 2), no significant impacts from the watering treatment were seen except that *C. acanthoides* survival to July, given emergence, was lower in watered plots; the magnitude of this effect was relatively large.

**Table 1:** *Carduus nutans* and *C. acanthoides* fall emergence and growth response to microsite characteristics.

		*Carduus nutans*		*Carduus acanthoides*	
	Explanatory variable	Coefficient	p- value	Coefficient	p- value
Emergence and fall survival in clipped plots	Intercept	–1.15			
	Area	0.64	0.00***		
	Watering	–0.55	0.02*		
	Interaction	0.55	0.02*		
Emergence and fall survival in fully disturbed plots	Intercept	–1.67			
	Area	1.14	0.00***		
	Watering	0.07	0.73		
	Interaction	–1.18	0.01**		
Emergence and fall survival	Intercept			–2.04	
	Disturbance Type			0.87	0.00***
	Area			0.19	0.02*
	Watering			–0.46	0.00**
Fall rosette size	Intercept	0.17		–0.12	
	Disturbance Type	–0.23	0.01**	0.49	0.000***
	Area	0.17	0.00***	0.17	0.000***
	Watering	0.12	0.08	0.03	0.67

* indicates significance of p≤0.05.

** indicates significance of p≤0.01.

*** indicates significance of p≤0.001.

Model results for fall measurements are shown in Table 1. Generalized linear mixed-effects models were fit, using likelihood ratio tests to determine whether model fit was improved by including interactions. Where higher order interactions were present (such as analyses of germination for *C. nutans),* Larger microsite area generally led to increased germination, survival and size. Rosette size was higher in clipped plots for *C. acanthoides* versus fully disturbed plots for *C. nutans.* Watering either had no effect or a negative effect;

**Table 2:** Post-overwintering response to microsite characteristics.

		*Carduus nutans*		*Carduus acanthoides*	
	Explanatory variable	Coefficient	p- value	Coefficient	p- value
Survival to July (given emergence)	Intercept			–0.21	
	Disturbance Type			0.50	0.09
	Area			0.33	0.02*
	Watering			–0.85	0.00**
Survival to July (given emergence) in clipped plots	Intercept	0.80			
	Area	0.44	0.02*		
	Watering	0.32	0.42		
Survival to July (given emergence)in fully disturbed plots	Intercept	0.28			
	Area	0.19	0.20		
	Watering	–0.45	0.11		
Proportion of thistles present which flowered	Intercept	–1.50		–3.39	
	Disturbance Type	0.83	0.02**	0.05	0.94
	Area	0.46	0.00**	0.90	0.00***
	Watering	0.14	0.66	0.12	0.82

* indicates significance of p≤0.05.

** indicates significance of p≤0.01.

*** indicates significance of p≤0.001.

Generalized linear mixed-effects models were fit, using likelihood ratio tests to determine whether model fit was improved by including interactions. Where higher order interactions were present (such as analyses of germination for *C. nutans),* separate models were fit on clipped and fully disturbed plots. Larger microsite area generally led to increased survival and flowering probabilities. Full disturbance led to higher proportions flowering for *C. nutans*. Watering typically had no significant effect; in one case, it had a negative impact on *C. acanthoides.*

## Discussion

Many biological invasions are associated with disturbance and the associated increase in resource availability [20], [34]. Our results show that disturbances that create gaps for emergence are critically important for the invasion success of *Carduus* thistles, and confirm that *C. nutans* and *C. acanthoides* emergence, survival and growth are strongly dependent on characteristics of their germination sites. In contrast, there is very poor emergence of these species in undisturbed vegetation [17], [21].

Microsite size appears to be particularly important, with larger microsites generally leading to better emergence and higher survival for both species. We did not observe a negative effect of very large gaps, suggesting that potential desiccation was not as critical during this study. The fact that larger size and higher flowering rates occurred in large microsites is in agreement with our observation of large, naturally occurring individuals of both species on highly disturbed areas with virtually no other vegetation, such as construction sites (E. Rauschert, pers. obs.). Such large individuals may be particularly important for spread, as tall height and lower surrounding vegetation leads to longer dispersal distances and hence greater invasion speed [35].

The distribution of gap sizes in old fields and pastures, which are typical habitats for these thistles, likely has important consequences for their ability to invade and persist in an area. Goldberg and Gross [1] studied gap characteristics in a mid-successional old field, and found that most gaps were small (less than 10 cm in size), and that animals were creating most of the new gaps. In New Zealand pastures with *C. nutans* infestations, gap sizes were also found to be small (mostly less than 10 cm), and there were fewer gaps in spring and autumn, when most emergence occurs [24]. Panetta and Wardle [24] suggest that hoof sized gaps may best promote emergence. This would be an unfortunate result for management, as grazing in wet pastures often creates gaps of such sizes. Wardle et al. [36] claim that some cover may be needed for a suitable microclimate for *C. nutans* emergence, as they observed low emergence in large bare ground plots. However, even in their experiments, individuals in the bare plots were more likely to flower in the first summer and grew larger than in plots with other vegetation.

For plants, the ability to access critical resources such as water is an important part of the invasion process. For example, *C. acanthoides* has been shown to be sensitive to desiccation during emergence and early growth [37]. Interestingly, additional water availability generally had a negative impact on emergence and survival in our study. This was unexpected, given most earlier work on these species, though one previous study found no effect of irrigation on thistle emergence and survival [21]. It is possible that watering may have washed some seeds deeper into the soil; both species have decreased emergence at increased soil depth, with *C. acanthoides* more vulnerable to this effect [16]. It is also possible that the addition of water initially benefited the existing vegetation more than the thistle seeds, an example of an indirect effect on competition [8]. Interestingly, the impact persisted longer than expected for *C. acanthoides,* where survival until July was negatively impacted. Davis and Pelsor [38] also suggest that early fluctuations in key resources, even only brief ones, may still have impacts a year later.

The differential response we observed to type of disturbance is intriguing. *Carduus acanthoides* had higher emergence rates in fully disturbed plots than in plots with above-ground disturbance (clipped) only. *Carduus nutans* had better performance generally in large clipped plots; it was less affected by the established below-ground environment than *C. acanthoides*. Seed size may be partially responsible for the differential response to disturbance; *C. acanthoides* seeds are considerably smaller than *C. nutans* seeds. It has been suggested that species with smaller seeds need larger gaps for establishment [39], to overcome the fact that they produce initially smaller seedlings; perhaps species with smaller seeds also benefit more from both above- and belowground disturbance. This effect is likely most important for early stages; by the following spring, both species were larger in initially fully disturbed microsites. However, such subtle differences in responses to microsite quality may play a role in invasion success, and should not be ignored.

We did see differences in the germination rates of the two cohorts, with higher germination rates in the second cohort. This may be due to maternal effects of the source populations on the quality of the seed produced in different years, as we have also observed large variation in germination rates of seeds from different years in greenhouse experiments (Rauschert, unpublished data). It is also possible that in 2005 in the fall, conditions were generally more favorable for *Carduus* germination and early seedling growth. For example, in September through November, 2004, there was twice as much rainfall as in 2005 (2788 mm vs. 1590 mm) [40], which may have benefitted the resident community more than the thistles [41]. Additionally, since we did not thin plots to one *Carduus* seedling if more than one plant germinated, there may have been some intraspecific competitive effects, but as 95% of plots had 2 or fewer germinations these effects were likely small.

It is worth noting that the dominant species present in the abandoned hayfield in central Pennsylvania, USA, were also non-native. Rauschert et al. (in revision) also observed that the species generally associated with *Carduus* thistles in central Pennsylvania are non-native; several are also associated with *Carduus* thistles in their native range [42]. In agricultural systems this is a common situation, as many weedy species were introduced along with crop species. Generally, invader-invader interactions, both positive [e.g. invasional meltdown, 43] and negative [e.g. invasional interference, 44,45], are likely to increase as the number of non-native species continues to rise.

Our results demonstrate that gap characteristics play an important role in emergence and establishment of these two species. Both microsite size and water availability affected establishment, but the type of disturbance generating the microsite also had a significant impact. These effects persisted well beyond these initial life stages. An understanding of the establishment and regeneration niche, and the role that disturbance plays at critical life stages, thus is an important part of any management plan for dealing with an invasion that has already occurred, and for maintaining community resistance to invasion. It is important to identify under what circumstances species are likely to grow well enough to establish large, persistent populations. In the case of these invasive plants, our results clearly suggest that minimizing the size and the intensity of disturbances, or reseeding of other vegetation, are most likely to hinder invasion success.
